# The Perils of the Post

**Published:** 1895-02-23

**Authors:** 


### THE PERILS OF THE POST.

Various means have been proposed for bottling up and transmitting by post samples of diphtherial membranes for bacteriological examination. The last is the simplest. The discharges intended for examination are to be received on a piece of white paper and immediately dried before the fire; the paper can then be folded with the discharge inside and transmitted by post in an ordinary envelope. When a culture is to be made it is only necessary to moisten the dried discharge with a little sterilised water, and in a few moments it is ready; in other words, the microbes are all alive again. The method certainly is handy, but it is not nice. That our letters are to be tossed about in the post-bag along with missives containing diphtheria germs wrapped in that fragile thing, an ordinary envelope, is certainly not reassuring.

